# Adverse effects of heavy cannabis use: even plants can harm the brain

**DOI:** 10.1097/j.pain.0000000000001963

**Published:** 2020-06-17

**Authors:** Lucia Sideli, Giulia Trotta, Edoardo Spinazzola, Caterina La Cascia, Marta Di Forti

**Affiliations:** a Department of Psychosis Studies, Institute of Psychiatry, Psychology and Neurosceince, King's College London, De Crespigny Park, Denmark Hill, London, United Kingdom; b Department of Neuroscience, Mental Health, and Sensory Organs (NeSMOS), Faculty of Medicine and Psychology, Sapienza University, Rome, Italy; c Department of Biomedicine, Neuroscience and Advanced Diagnostic, Palermo University, Palermo, Italy; d Social, Genetic and Developmental Psychiatry Centre, Institute of Psychiatry, Psychology and Neuroscience, King's College London, London, United Kingdom; e National Institute for Health Research (NIHR) Mental Health Biomedical Research Centre at South London and Maudsley NHS Foundation Trust and King's College London, London, United Kingdom; f South London and Maudsley NHS Mental Health Foundation Trust, London, United Kingdom

## 1. Introduction

The spread of laws legalising cannabis for medicinal or recreational use has been accompanied by more relaxed attitudes towards cannabis. Data from the United States show that in states that have legalised cannabis, prevalence of daily, weekly, and monthly cannabis use was 11.3%, 18.3%, and 25.0% respectively, whereas in countries where it is still illegal, it was lower (7.4%, 11.6%, and 16.8% respectively).^46^ Evidence indicates a trend of increase among adolescents,^58^ a particular vulnerable category for the initiation of substance use.^114^ In parallel, we have seen the concentration of THC (Δ-9-tetrahydrocannabinol) in the cannabis sold both in the United States and in Europe rising and those types of cannabis with high THC,^35^ and a corresponding decrease of cannabidiol (CBD) content,^17,35,95^ becoming more widely available. Most commonly, cannabis is used for its enjoyable effects, the “high” feeling.^69^ In addition, in those countries where its use has been legalised, many people smoke cannabis for medical use, anxiety, depression, and pain relief,^71^ with those suffering from chronic pain being at higher risk of developing cannabis use disorder (CUD).^57^

This review aims to challenge the widespread view that cannabis being a “plant” does not carry adverse effects, and review the evidence concerning the effects of cannabis use on mental health and cognition.

## 2. Understanding cannabis

The plant *Cannabis sativa* contains more than 100 different psychoactive ingredients,^33,53,74^ but the most widely studied are THC (Δ-9-tetrahydrocannabinol) and CBD (cannabidiol),^36,74,84^ synthesised from the same precursor, cannabigerol. Therefore, types of cannabis with high concentrations of THC produce low CBD and vice versa.^23^

**Figure 1.**
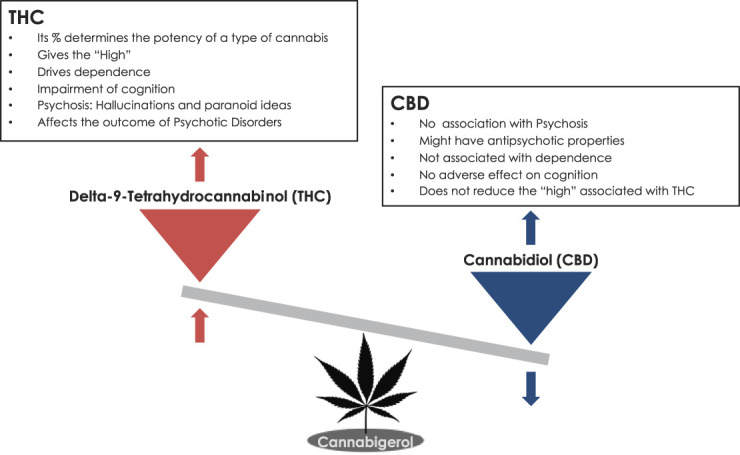
This diagram graphically illustrated first, the differential and in fact opposite psychiatric and cognitive effects of THC and of CBD, and second, how both compounds derive from the same precursor. Therefore if, for instance, a *Cannabis sativa* plant is genetically driven to the production of high quantity of THC, it will only be capable to synthesise small quantities of CBD.^10^

THC is a partial agonist at both the cannabinoid receptors (CB) CB_2_ R and CB_1_ R, the latter highly abundant in the brain.^64, 83, 97^ THC is responsible for most of the enjoyable and adverse effects that commonly arise after acute and regular cannabis use.^8,24,116^ Conversely, CBD is a negative allosteric modulator at the CB_1_R,^76^ whose effects are distinct and, in many cases, opposite of those observed with THC. CBD does not induce euphoria but may exert anxiolytic, antiepileptic, anti-inflammatory, and analgesic properties Figure 1.^9,10,22,91,99,100,108^

## 3. Cannabis intoxication and dependence

### 3.1. Acute adverse effects

Cannabis use can lead to intoxication, defined as a series of “clinically significant problematic behavioural or psychological changes (eg, impaired motor coordination, euphoria, anxiety, sensation of slowed time, impaired judgment, and social withdrawal) that develop during, or shortly after, cannabis use.”^2^ The experience of feeling “high” after cannabis use is variable and subjective,^48^ depending on the dose, the environment, and previous experience and expectations of the drug user.^101^ The most common acute adverse effects are panic attacks and other forms of anxiety, mostly reported by naive users.^50,51^ Because cannabis impairs psychomotor skills, reaction time, and motor coordination, its use leads to increased risk of motor vehicle accidents.^6,98^ Current cannabis users have higher rates of hospitalization for injury from all causes than former cannabis users or nonusers.^41^

### 3.2. Cannabis use disorders

DSM-5 CUDs is now a single diagnostic entity including abuse and dependence^3^; it is prevalent (2.54% 12-month prevalence among U.S. adults), associated with comorbidity and disability, and largely untreated.^56^ Moreover, CUD often presents comorbidly with psychotic disorders.^59^ Preclinical studies support the evidence that exposure to THC leads to dependence.^1,16,19^ Cannabis can induce tolerance in humans^60,65^ and dependence.^16^ Because cannabis is eliminated slowly from the body, withdrawal symptoms are generally mild. Nevertheless, discontinuation of long-term frequent cannabis use can induce anger, decreased appetite, irritability, nervousness, restlessness, and sleep difficulties,^13,79^ suggesting that the alleviation of abstinence symptoms contributes to the maintenance of daily cannabis use.^52^ Recent evidence, from the Netherlands, has shown an association between changes over time in average THC content in the type of cannabis available and rates of admission to specialist cannabis treatment units.^37^

## 4. Cannabis and psychosis

### 4.1. Epidemiological evidence

Cross-sectional and prospective studies demonstrate a causal link between cannabis use and psychotic disorder, with greater risk for cannabis users, compared to nonusers.^73,85,88,89^ The most recent meta-analysis reported a pooled odds ratio (OR) = 3.90 (95% confidence interval [CI] 2.84-5.34) and shows a dose–response association between cannabis use and psychosis outcomes.^82^ Furthermore, findings from general population studies indicate, even after statistical adjustment for other known psychosis risk factors, a strong association between cannabis use and psychotic symptoms, and especially paranoia, beyond the clinical disorder.^34,92,95,102^

### 4.2. Harmful patterns of cannabis use

Data from South London showed that use of high-potency cannabis (skunk-type average THC% = 14%), compared to never use, was associated with a 3-fold risk (OR = 2.92, 95% CI 1.52-3.45), which increased to 5-fold when the use was daily (OR = 5.4, 95% CI 2.81-11.31), of developing psychotic disorders; hash-like cannabis use carried no additional risk compared to never use—likely because of ratio of THC: CBD = 1 ratio in most of the London hash available at the time of the study.^28,29^ Consistently, survey data from general population samples suggest that use of cannabis with high CBD content is associated with fewer psychotic experiences.^10,87^ Morgan and Curran^87^ showed that healthy volunteers with hair traces of THC and cannabidiol reported less schizophrenia-like symptoms than those with only THC in their hair traces of THC.^87^

More recently, data from the EUGEI study, a large multicentre European collaboration,^40^ confirmed that those who use daily types of cannabis with THC content = >10% are 5 times (OR = 4.8; 95% CI 2.5-6.3) more likely to suffer from psychotic disorder than never users^30^; a risk that was greater OR = 9.43 (95% CI 6.2-19.6) in Amsterdam where popular types of cannabis such as Nederhasj and Nederwiet have THC contents that can reach 67% and 22%, respectively.^30,90^ Moreover, first episode psychosis patients (FEP), who used high-potency cannabis daily experienced, at their illness onset, more prominent positive symptoms (eg, delusions and hallucinations) and, in particular, paranoia.^96^

### 4.3. Cannabis use and age of onset of psychotic disorders

A meta-analysis by Large et al.^77^ reported that subjects who used cannabis experienced, on average, their illness onset 3 years earlier compared to never users, a significantly higher effect than for use of other drugs including alcohol. Another study showed that if subjects had used high-potency cannabis daily, their illness onset was, on average, 6 years earlier^31^ compared to never users.

### 4.4. Heavy cannabis use and rates of psychotic disorders

Boydell et al.^11^ claimed that cannabis consumption could impact on the incidence of schizophrenia in those areas where the prevalence of cannabis use is high. The first, clear evidence of the impact of cannabis use on rates of psychotic disorder comes from the EUGEI study. Across 11 European sites, the incident rates for psychotic disorder—adjusted for age, sex, and ethnicity—were positively correlated with the prevalence of use of high-potency cannabis (*r* = 0.7; *P* = 0.0286) and, independently, with the prevalence of daily use (*r* = 0.8; *P* = 0.0109) among the controls representative of the local populations.^30^ These findings suggest that where daily cannabis use and use of high-potency cannabis is prevalent in the general population, there are more new cases of psychotic disorders. Indeed, the study shows that 12% of patients with FEP across Europe can be attributed to high-potency cannabis use, rising to 30% in London and 50% in Amsterdam.^30^ If high-potency cannabis were no longer available, incidence in London would drop from 46 × 100,000 to 32 × 100,000 person-years, even after taking into account age, sex, and migration. Further independent evidence comes from Portugal that has registered a steady increase in the rate of hospital admissions for psychotic disorders with comorbid CUD.^45^ Similar data were reported in Denmark.^59^ Both countries have seen a rise in the potency of available cannabis over the same period.^35,111^

### 4.5. Reverse causality and self-medication hypotheses

The self-medication hypothesis suggests that patients with psychotic disorders use cannabis to seek relief from their symptoms.^4,43,106^ The Christchurch Health & Development birth cohort study showed that although cannabis use was associated with increasing psychotic symptoms, the experience of psychotic symptoms inhibits rather than encourages subsequent cannabis use.^25,26^ Moreover, findings from the Dunedin birth cohort indicated a specific temporal link between cannabis use and onset of psychosis outcomes. Cannabis use at 15 years of age was associated with a 4-fold increase in risk for schizophreniform psychosis at 26 years of age (OR = 4.50, 95% CI 1.11-18.21), compared to never use. This association remained present (OR = 3.12; 95% CI 0.73-13.29) after excluding those participants who at 11 years of age had reported psychotic symptoms, although failed to reach significance because of reduced power.^5^

More recently, Mendelian randomization investigated the relationship between cannabis use and randomly assorted genetic variants that are associated with psychosis, which were used as proxy for psychosis itself. Mendelian randomization studies have suggested that cannabis use initiation is partly explained by common genetic variants associated with risk of schizophrenia, thus proposing a direction of causality from schizophrenia genes to cannabis use (ie, reverse causality) rather than from cannabis use to schizophrenia and other psychosis.^39,93,115^ By contrast, findings from the EUGEI study showed that (1) genetic summary score for schizophrenia (polygenic risk score [PRS]) did not predict the propensity to initiate cannabis use, (2) how frequently someone uses it, and (3) the potency of the cannabis used. On the contrary, heavy cannabis use increased the risk for psychotic disorders independent of the individual's schizophrenia PRS. For instance, daily users of high-potency cannabis (THC = >10%) had a 5-fold increase (OR = 5.4; 95% CI 3.21-10.63) in their risk for psychotic disorders, even after controlling for the schizophrenia PRS.^32^

### 4.6. Course and outcome of psychosis

A meta-analysis indicates that patients with a psychotic disorder who continue to use cannabis after their illness onset experience a worse clinical and functional outcome than those who stop.^103^ Data from a 2-year follow-up study showed that FEP who used high-potency cannabis daily over the follow-up period were 3 times more likely to relapse (OR = 3.28; 95% CI 1.22-9.18), experienced more relapses (incidence rate ratio 1.77; 95% CI 0.96-3.25), and received more intense psychiatric care (OR 3.16; 95% CI 1.26-8.09), compared to those who stopped.^18,104,105^

Some evidence begin to suggest that individuals at ultra-high risk for psychosis have higher rates of CUDs^14^ and, conversely, patients with CUDs are more likely to transition to psychosis.^72^

### 4.7. Self-reported measures of cannabis use

All the above epidemiological studies rely on self-reported current and/or lifetime information on cannabis use, not validated by biological measures (eg, urine, blood, and hair samples). This is often considered a limitation. Nevertheless, although biological measures can provide valid and reliable measures of current use, they cannot provide data on use over time. Indeed, studies that analysed both self-reported information and laboratory data indicated that cannabis users are reliable in reporting how frequently they use and the type they used.^21,36^

### 4.8. Human experimental studies

Administration of cannabis and THC has shown to precipitate, with a dose–response relationship, the onset of transient positive psychotic symptoms (eg, ideas of reference, paranoid delusions, hallucinations, depersonalization, or derealization) and, to a less extent, negative symptoms (eg, blunted affect) in healthy volunteers and to temporary exacerbated psychotic symptoms in schizophrenia patients.^88,110^ Furthermore, in healthy volunteers, administration of CBD before the THC was found to ameliorate the psychotogenic effects of THC.^88,110^

### 4.9. Vulnerable groups

The Dunedin study was the first to indicate adolescents as a group particularly vulnerable to the psychotogenic effect of cannabis use.^5^ Since then, other studies have reported an association between early cannabis initiation and greater risk for psychosis.^15^ It remains unclear if this association reflects (1) a longer duration of exposure (eg, earlier start, longer use) or (2) the vulnerability of a developing brain.^88, 117^

Subjects with a family history of psychotic disorders have a greater sensitivity to the psychotogenic effect of cannabis^66^ and if they develop a cannabis-induced psychotic disorder, they are more likely to transition to schizophrenia.^68^ Furthermore, individuals who have a high schizophrenia PRS and use cannabis heavily are at higher risk for psychosis than those who either carry a high schizophrenia PRS or smoke cannabis heavily.^32^ A recent study described an additive interaction between schizophrenia PRS and cannabis use,^32,49^ with no evidence that genetic liability increases the risk for cannabis use.

Another potentially vulnerable population might be represented by individuals exposed to childhood adversity, which may enhance the psychotogenic effect of cannabis, through sensitization. Several studies observed that the joined effect of early trauma and cannabis use on psychosis was greater than their independent effect,^54,62,63,70^ but the findings were not fully consistent.^7,86,112^

## 5. Cannabis and cognitive impairment

Two meta-analyses^47,107^ described a modest residual cannabis-related impairment in measures of both overall and specific cognitive functions after 12 hours to 25 days of abstinence, with no residual cognitive impairment after 25 days of abstinence.^107^ Another meta-analysis including samples of adolescent and young adults found a modest overall negative effect (d = −0.25; 95% CI −0.32 to −0.17) of cannabis use on cognition and no residual effect (d = −0.08; 95% CI −0.22 to 0.07).^109^

In young adults, chronic cannabis use most commonly affects immediate recall and verbal reasoning^12,38,118^ but not spatial working memory; however, the latter is affected in adolescents,^55^ suggestive of a differential effect on the developing brain. Both in adolescents and adult users, attention is impaired during cannabis intoxication and persists for several weeks.^61^ Executive function domains (eg, inhibition, problem solving) are differently affected by acute or chronic cannabis use, and it is not clear how likely impairments are to persist after abstinence.^27,75^

## 6. Cannabis and bipolar disorder

Regular cannabis use is associated to about a 3-fold risk (OR = 2.97, 95% CI 1.8-4.9) of developing a manic episode, with some evidence of dose–response relationship between frequency of use and risk for mania.^42,81,94^ Furthermore, continued cannabis use and CUDs increases the severity of manic and psychotic symptoms and facilitates a rapid-cycling course of bipolar disorder.^42,80^

## 7. Cannabis, depression, and anxiety

Regular cannabis use is associated with lack of motivation for naturally rewarding activities, which is a core feature of depressive disorders.^117^ Systematic reviews indicate that cannabis use leads to a modest increase in the risk for depression (OR = 1.49, 95% CI 1.15-1.94),^85^ which becomes slightly greater (OR = 1.62, 95% CI 1.21-2.16)^78^ for frequent cannabis use. Furthermore, those who start using cannabis at age = <15 years are at greater risk for suicidal ideation (OR = 1.50, 95% CI 1.11-2.03) and suicidal behaviours (OR = 3.46, 95% CI 1.53-7.84) both in general population and clinical samples.^44^

Evidence for a weaker association between cannabis use and anxiety disorders comes from a meta-analysis, estimating ORs from 1.15 (95% CI 1.03-1.29)^113^ to 1.24 (95% CI 1.06-1.45).^67^ Nevertheless, more longitudinal studies are needed to clarify the direction of the association between cannabis use depression and anxiety,^78,113^ to examine the role of self-medication.^20,119^

## 8. Conclusions

All prescribed and recreational drugs have adverse effects, even those coming from plants, fruits, and flowers as we have learnt from the use of tobacco, alcohol, and opium. Cannabis is not an exception (Tables 1 and 2). Therefore, at a time of changes in the laws concerning cannabis use, it is of clinical and public health importance to provide evidence-based and clear information on what we know concerning (1) the acute and persistent adverse effects and (2) how to screen for those individuals more susceptible to experience them when cannabis is used recreationally or medicinally.

**Table 1:** Summary of meta-analyses reporting adverse effects associated with cannabis use.

Adverse effect	Participants	Studies	Main findings	Estimate
Psychosis				
Marconi et al.^18^	66,816 individuals from 10 studies	Random-effects meta-analysis on risk of psychosis	High levels of cannabis use increase the risk of psychotic outcomes with a dose–response relationship	OR = 3.9, 95% CI [2.84-5.34]
Large et al.^77^	8167 substance using patients from 83 studies	Random-effects meta-analysis on age at onset of psychosis	Relationship between cannabis use and earlier onset of psychotic illness	ES = −2.70, 95% CI [−0.53 to −0.30]
Schoeler et al.^103^	16,565 individuals from 24 studies	Random-effects meta-analysis on clinical outcomes of psychosis	Continued cannabis use after onset of psychosis predicts adverse outcome than for nonusers	d = 0.31, 95% CI [0.04-0.57]
Bipolar				
Gibbs et al.^42^	2391 individuals from 6 studies	Random-effects meta-analysis	Association between cannabis use and both the exacerbation of manic symptoms in those with previously diagnosed bipolar disorder and new-onset manic symptoms	OR = 2.97, 95% CI [1.8-4.9]
Depression				
Gobbi et al.^44^	22,317 individuals from 11 studies	Random-effects meta-analysis	Cannabis consumption in adolescence is associated with increased risk of developing depression in young adulthood	OR = 1.37, 95% CI [1.16-1.62]
Lev-Ran et al.^78^	76,058 individuals from 14 studies	Random-effects meta-analysis	Heavy cannabis use may be associated with an increased risk for developing depressive disorders	OR = 1.62, 95% CI [1.21-2.16]
Anxiety				
Gobbi et al.^44^	22,317 individuals from 11 studies	Random-effects meta-analysis	No evidence of an association with anxiety	OR = 1.18, 95% CI [0.84-1.67]
Twomey et al.^113^	58,538 individuals from 10 studies	Random-effects meta-analysis	Cannabis use is no more than a minor risk factor for the development of elevated anxiety symptoms in the general population	aOR = 1.08, 95% CI [0.94-1.23]
Cognition				
Grant et al.^47^	623 cannabis users and 409 minimal or non-cannabis users from 11 studies	Fixed-effects meta-analysis	There might be decrements in the ability to learn and remember new information in chronic users, whereas other cognitive abilities are unaffected	Learning ES = −0.21, 99% CI [−0.39 to 0.02] Forgetting/retrieval ES = −0.27, 99% CI [−0.49 to −0.04]
Schreiner et al.^107^	1010 current or former cannabis users and 837 controls with no or limited cannabis use from 33 studies	Random-effects meta-analysis	A small negative residual effect of cannabis use on overall cognitive performance, no evidence of lasting residual effect	Overall cognitive performance ES = −0.29, 95% CI [−0.46 to −0.12] Lasting residual effect ES = −0.12, 95% CI [−0.32 to 0.07]

This table illustrates the findings from meta-analyses that report an association between several mental health outcomes, cognition, and cannabis use.

CI, confidence interval; ES, effect size; OR, odds ratio.

**Table 2:** Summary of the mental health and cognitive acute and persistent adverse effects associated with cannabis use.

Adverse effects	Acute	Persistent	Pattern of cannabis use	Vulnerable individuals
Onset of psychotic symptoms	+++	+++	1. Dose–response relationship (% THC) 2. CBD ameliorates THC effects	1. Family history of psychosis 2. High polygenic risk score for schizophrenia 3. Adolescents (age at first use = <15 y)
Worsening of psychotic symptoms	+++	+++	1. Daily use of high-potency cannabis	NAD
Onset of mania	++−	++−	1. Dose–response relationship with frequency of use	NAD
Worsening of manic symptoms	++−	++−	1. Cannabis use disorder	NAD
Depression	+−−	+−−	1. Dose–response relationship with frequency of use	NAD
Anxiety	++−	+−−	1. Dose–response relationship with frequency of use	NAD
Suicidal ideation	+−−	+−−	NAD	Adolescents (age at first use = <15 y)
Dependence (CUD)	NA	+++	1. Dose–response relationship with THC content (high-potency cannabis and daily use) 2. CBD ameliorates THC effects	NAD
Withdrawal symptoms	NA	+++	Long-term frequent use	NAD
Psychomotor skills, reaction time, and motor coordination	+++	NAD	1. Dose–response relationship (% THC)	Cannabis-naive individuals
Verbal learning and memory	+++	+−−	1. Dose–response relationship (% THC) 2. CBD ameliorates THC effects	
Working memory	++−	+−−	1. Dose–response relationship (% THC) 2. CBD ameliorates THC effects	
Spatial working memory	++−	+−	1. Dose–response relationship (% THC)	Adolescents
Attention	+++	++−	1. Dose–response relationship (% THC) 2. CBD ameliorates THC effects	Adolescents
Executive function domains	++−	+−−	NAD	Adolescents

+++, strong consistent evidence; ++−, modest evidence; +−−, weak or inconsistent evidence; CBD, cannabidiol; CUD, cannabis use disorder; NAD, not available data and/or not investigated; NA, not applicable; THC, delta 9-tetrahydrocannabinol.

This table lists (1) the acute (2) and/or persistent effects that have been reported after cannabis use and the strength of the related evidence, (3) the patterns of cannabis use mostly associated with each adverse effects, and (4) the individuals reported to be the most vulnerable to experience them.

## Conflict of interest statement

M. Di Forti reports personal fees from Janssen, outside the submitted work. The remaining authors have no conflict of interest to declare.
